# Persistence of disseminated tumor cells after neoadjuvant treatment for locally advanced breast cancer predicts poor survival

**DOI:** 10.1186/bcr3242

**Published:** 2012-08-14

**Authors:** Randi R Mathiesen, Elin Borgen, Anne Renolen, Erik Løkkevik, Jahn M Nesland, Gun Anker, Bjørn Østenstad, Steinar Lundgren, Terje Risberg, Ingvil Mjaaland, Gunnar Kvalheim, Per Eystein Lønning, Bjørn Naume

**Affiliations:** 1 Department of Genetics, Institute for Cancer Research, Oslo University Hospital, The Radium Hospital, Ullernchausseen 70, Oslo, 0310, Norway; 2 Division of Surgery and Cancer Medicine, Department of Oncology, Oslo University Hospital, Ullernchausseen 70, Oslo, 0310, Norway; 3 Department of Pathology, Oslo University Hospital, The Radium Hospital, Ullernchausseen 70, Oslo, 0310, Norway; 4 Section of Oncology, Institute of Medicine, University of Bergen, Jonas Lies vei 65, Bergen, 5020, Norway; 5 Department of Oncology, Haukeland University Hospital, Jonas Lies vei 65, Bergen, 5020, Norway; 6 Department of Oncology, St. Olav University Hospital, Olav Kyrres gate 17, Trondheim, 7006, Norway; 7 Department of Cancer Research and Molecular Medicine, Norwegian University of Science and Technology, Hogskoleringen 1, Trondheim, 7491, Norway; 8 Department of Oncology, University Hospital of Northern Norway and Institute of Clinical Medicine, University of Tromsø, Sykehusvegen 38, Tromsø, 9037, Norway; 9 Division of Hematology and Oncology, Stavanger University Hospital, Armauer Hansens vei 20, Stavanger, 4011, Norway; 10 Institute of Clinical medicine, Faculty of Medicine, University of Oslo, Kirkeveien 166, Oslo, 0450, Norway

## Abstract

**Introduction:**

Presence of disseminated tumor cells (DTCs) in bone marrow (BM) and circulating tumor cells (CTC) in peripheral blood (PB) predicts reduced survival in early breast cancer. The aim of this study was to determine the presence of and alterations in DTC- and CTC-status in locally advanced breast cancer patients undergoing neoadjuvant chemotherapy (NACT) and to evaluate their prognostic impact.

**Methods:**

Bone marrow and peripheral blood were collected before NACT (BM1: *n *= 231/PB1: *n *= 219), at surgery (BM2: *n *= 69/PB2: *n *= 71), and after 12 months from start of NACT (BM3: *n *= 162/PB3: *n *= 141). Patients were included from 1997 to 2003 and followed until 2009 (or ten years follow-up). DTC- and CTC-status were determined by morphological evaluation of immunocytochemically detected cytokeratin-positive cells. The prognostic significance of DTCs/CTCs was assessed by univariate and multivariate Cox-regression analyses.

**Results:**

Before NACT, DTCs and CTCs were detected in 21.2% and 4.9% of the patients, respectively. At surgery, 15.9% and 1.4% had DTC- and CTC-presence, compared to 26.5% and 4.3% at 12 months from start of NACT. Of patients for whom DTC results both before NACT and at 12 months were available, concordant results were observed in 68%, and 14 out of 65 had positive DTC-status at both time points. Presence of ≥ 1 DTC 12 months from start of NACT, but not at other time points, predicted reduced disease-free survival (DFS; HR 2.3, p = 0.003), breast cancer-specific survival (BCSS; HR 3.0, p < 0.001) and overall survival (OS; HR 2.8, p < 0.001). Before NACT, presence of ≥ 3 DTCs was also associated with unfavorable outcome, and reduced BCSS was observed for CTC-positive patients (HR 2.2, p = 0.046). In multivariate analysis, DTC status (</≥ 1 DTC) at 12 months after start of NACT remained as a prognostic factor for both DFS (HR 2.2, p = 0.005), BCSS (HR 2.6, p = 0.002) and OS (HR 2.6, p = 0.002). The survival for patients with change in DTC-status was determined by the DTC-status at 12 months.

**Conclusion:**

Presence of DTCs after NACT indicated high risk for relapse and death, irrespective of the DTC-status before treatment. The results supports the potential use of DTC analysis as a monitoring tool during follow up, for selection of patients to secondary treatment intervention within clinical trials.

## Introduction

Despite earlier detection of breast cancer, a substantial number of patients are diagnosed with locally advanced disease. Larger tumor sizes, lymph node spread, and unfavorable tumor biology all contribute to higher risk of micrometastases at the time of diagnosis [1]. Increased use of systemic treatment, based on combined use of prognostic and predictive factors, has resulted in improved survival for all stages of early breast cancer [2]. However, there is still need for additional markers to guide clinical decision making.

During the last decade, disseminated tumor cells (DTCs) in the bone marrow (BM) at the time of diagnosis have been shown to be an independent prognostic factor in early stage breast cancer [3]. In addition, persistence of DTCs after a median relapse-free follow-up interval of three years indicated an elevated risk of relapse [4,5]. In contrast to primary tumor markers, analysis of DTCs may be used in the follow-up situation as a surrogate marker to identify patients with a poor response to adjuvant therapy. However, only a few smaller studies have tested the significance of DTCs early after completion of chemotherapy, with inconsistent results [6,7]. In metastatic breast cancer, detection and persistence (during therapy) of circulating tumor cells (CTCs) identifies patients with an especially poor prognosis [8-12]. More recently, CTCs have been shown to be associated with a worse prognosis also in patients with non-metastatic breast cancer [13-17].

The aim of neoadjuvant chemotherapy (NACT) is to reduce tumor size prior to surgery and eradicate micrometastases. Today, the evaluation of the effect of this therapy is based on the assessment of local tumor response measured by clinical assessment, imaging modalities and histopathological examination after breast surgery. While a pathological complete response (pCR) after neoadjuvant treatment is associated with improved long-term prognosis [18], at least for certain subgroups of breast cancer [19,20], there is still a need for novel tools and surrogate markers for assessment of the adjuvant treatment efficacy. Analysis of the fate of DTCs and CTCs during and after treatment can give information on the presence and load of minimal residual disease at distant sites and indicate the need for optimized treatment.

The primary aim of this study (The Neotax Study) was to identify markers predicting drug resistance to epirubicin versus paclitaxel monotherapy in locally advanced breast cancer. The results related to antitumor response, disease-free survival (DFS) and breast cancer-specific survival (BCSS) have been published earlier [21,22]. In a separate, predefined substudy, we explored the changes and prognostic impact of DTCs and CTCs at different time points during treatment and follow-up, and correlated these findings to other clinicopathological parameters.

## Materials and methods

### Patients

The Neotax Study enrolled a total of 260 patients with stage III/IV breast cancer on an intention-to-treat basis. The study was a national study including participation by all the Norwegian University hospitals [21,22]. Out of these, a total of 236 patients signed separate informed consent forms for participation in the micrometastasis substudy of disseminated tumor cells and circulating tumor cells. The study protocol was approved by the Regional Ethical Committee (Norwegian Health Region III). The recruitment period was between November 1997 and December 2003. The patients were followed until October 2009, or maximally for 10 years or until death.

Patients with locally advanced non-inflammatory breast cancer (cT3-4 and/or cN2) were included in the study. The routine diagnostic workup included mammography, a surgical biopsy of the primary tumor, radiographs of the chest, pelvic area and lumbar spine, ECG, liver ultrasound, bone scintigraphy, blood samples and clinical examination including caliper measurement of the primary tumor. This was performed before starting the neoadjuvant chemotherapy. The protocol allowed inclusion of patients with limited distant metastases (that is, locoregional metastases, limited skeletal metastases with alkaline phosphatase (ALP) ≤ double the upper normal limit or solitary lung or liver metastases for whom, in the opinion of the investigator, their local tumor represented the major therapeutic challenge). BM aspiration was not performed if metastastasis was present in the iliac crest. The patients were followed at each of the hospital's outpatient departments with clinical examination, mammography, blood samples and chest radiograph on an annual basis.

### Treatment protocol

The Neotax study was an open-labeled multicenter study in which patients were randomly allocated to treatment with paclitaxel 200 mg/m^2 ^(*n *= 129) or epirubicin 90 mg/m^2 ^(*n *= 131) administered every third week. Of the patients included in the DTC/CTC substudy, 115 were treated with paclitaxel and 121 with epirubicin. The effects of treatment on the primary tumor were graded by the UICC system [23] and not the newer RECIST criteria [24] since the implementation of the protocol was in October 1997. Thus, responses were classified as complete response (CR), that is, complete disappearance of all tumor lesions; partial response (PR), that is, ≥ 50% reduction in the sum of all tumor lesions calculated for each as the product of the largest diameter and the one perpendicular to it; progressive disease (PD), that is, increase in the diameter product of any individual tumor lesion by ≥ 25%), or stable disease (SD), that is, anything between PR and PD). A crossover between the treatment arms was performed if there was no response (that is, SD or PD after three to four courses). Chemotherapy was followed by mastectomy and level 1 and 2 axillary clearance. After surgery, the patients received locoregional radiotherapy against the chest wall, ipsilateral axilla and supraclavicular fossa (48 or 50 Gy depending on current local practice). In case of tumor infiltration at the edge of the specimen, a boost up to 10 Gy was applied to the tumor bed in accordance with general practice. All patients with estrogen receptor (ER)-positive tumors (*n *= 123) were given tamoxifen 20 mg × 1 for five years, except for postmenopausal women who were on tamoxifen treatment up to mid-2004. According to the change in the Norwegian Breast Cancer Group guidelines for adjuvant endocrine therapy, these patients all switched to three years treatment of aromatase-inhibitor after completing two to five years of tamoxifen treatment [25].

### Bone marrow aspiration and peripheral blood collection

BM aspiration (bilateral iliac crest aspirates) and peripheral blood (PB) samples were obtained for analysis of DTCs/CTCs at three time points: prior to commencement of chemotherapy (BM1, *n *= 231 and PB1, *n *= 219), on the day of mastectomy (BM2, *n *= 69 and PB2, *n *= 71), and 12 months after the day of randomization (BM3, *n *= 162 and PB3, *n *= 141). The study was run by oncologists, and surgeons were encouraged to perform BM aspirations at surgery (BM2). Logistical reasons and variability in the surgeon's motivations or skills was the reason for the low number of samples collected at surgery. To avoid any influence on the CTC or DTC results from possible tumor cell-shedding during surgery, BM and blood samplings were performed prior to surgery.

### Analysis of primary tumor and axillary lymph nodes

The initial (pretreatment) surgical biopsy of the primary tumor and primary tumor or axillary lymph nodes resected at final surgery were processed on a routine diagnostic basis. Histological tumor type, grade, hormone receptor status, tumor size at the time of surgery, in addition to lymph node involvement, were analyzed. Tumors were analyzed for estrogen receptors (ER) and progesterone receptors (PgR) by immunohistochemistry and were considered positive if > 10% of tumor cells stained positive with anti-ER- and/or anti-PgR antibodies, according to standard procedure in Norway at the time of the study. HER2 status is unknown because this was not a part of the routine analyses at the time of the study.

### BM aspiration and PB sampling

For each sampling, a total of 30 to 40 ml of BM was bilaterally aspirated from the posterior iliacal crest under local anesthetic, and 50 ml of peripheral blood was collected from each patient. The processing and analysis of DTCs and CTCs have been previously described [26]. Briefly, after separation by Ficoll-Hypaque density centrifugation, mononuclear cells (MNC) were collected and cytospins prepared (8 slides with 0.5 × 10^6 ^MNC/slide). Four slides (2 × 10^6 ^MNC) were incubated with the anticytokeratin monoclonal antibodies (mAbs) AE1 and AE3 (Sanbio, Uden, the Netherlands). For each sample, the same number of slides was incubated with an irrelevant monoclonal antibody of the same immunoglobulin isotype (MOPC21, Sigma-Aldrich, Saint Louis, Missouri, USA), as a negative control. For detection of mAb-bound cells, the standard alkaline phosphate/antialkaline phosphatase (APAAP) method [27] with New Fuchsin as chromogen, was used. The slides were counterstained with heamotoxylin to visualize the morphology of the nucleus.

### Detection of CTCs/DTCs

The cytospins were manually screened with a light microscope (x 10 lens) by a pathologist. The immunostained cells that met certain predefined morphological criteria [28], were scored as DTCs or CTCs. Patient samples harboring one or more cells that were characterized as malignant, were considered positive. Samples harboring cells scored as tumor cells in both AE1AE3-incubated slides and in the corresponding negative control slides were considered as not evaluable and excluded from conclusion.

### Statistical analysis

Breast cancer-specific survival (BCSS) and overall survival (OS) was measured from the date of randomization to death from breast cancer or any death, otherwise censored at the time of the last follow-up visit or at non-cancer related death (for BCSS). Disease-free survival (DFS) was measured the same way, according to the presence of locoregional or systemic relapse, and was only analyzed in patients without metastases (M0 patients). Kaplan-Meier survival curves for time to distant recurrences and breast cancer-specific death were constructed. *P*-values were computed by the log-rank test. Cox proportional hazard regression was used for univariate and multivariate analysis of prognostic impact of relevant variables. For statistical analysis, SPSS (PASW Version 18; SPSS, Chicago, Illinois, USA) software was used.

## Results

### Patient characteristics and detection of DTCs and CTCs

**Figure 1:**
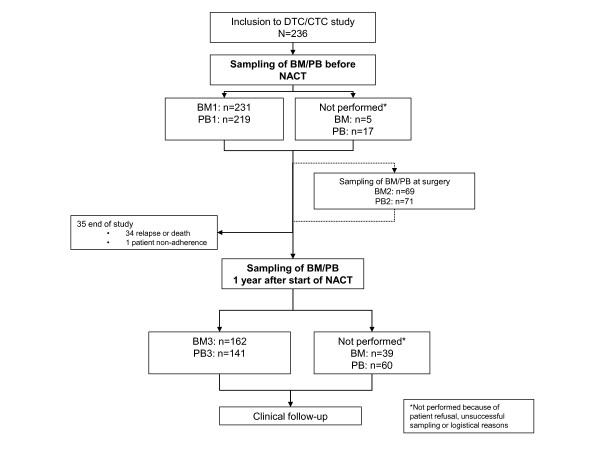
**Study overview of patients and samples at the different time points**. Bone marrow (BM) aspiration and peripheral blood (PB) sampling performed pretreatment (BM1 and PB1), at surgery (BM2 and PB2), and 12 months after start of neoadjuvant treatment (BM3 and PB3). NACT, neoadjuvant chemotherapy.

A total of 260 patients were included in the study on an intention-to-treat basis. Of these, 236 accepted participation in the micrometastasis substudy (Figure 1). Descriptive clinical, histopathological and CTC/DTC data of the patients enrolled are presented in Table 1. The median age at diagnosis was 51 years. The immunocytochemical (ICC) analysis disclosed ≥ 1 DTC/2 × 10^6 ^MNC in 21.2% of the patients before neoadjuvant chemotherapy (NACT) (at BM1). After 12 months from the start of NACT (BM3), 26.5% of the patients were DTC-positive. Sixty-nine patients had BM aspiration performed at surgery (BM2), with presence of DTCs in eleven patients (15.9%). No association was found between DTC status and primary tumor characteristics. Of those that had BM aspiration at BM1 and BM3, concordant results were observed in 68% of the patients. Fourteen of the 65 patients who were positive at BM1 and/or BM3 (22%), had presence of DTC at both occasions (Table 1). The incidence of CTCs before NACT was 4.9% compared to 1.4% and 4.3% on sampling at PB2 and PB3, respectively.

**Table 1:** Clinicopathological data and DTC status at BM1 and BM3

Variable	Number of patients (N)	DTC+ at BM1 n/number assessed^d ^(%)	*P-*value	DTC+ at BM3 n/number assessed^d ^(%)	*P-*value
**Total number**	**236**	**49/231 (21.2)**		**43/162 (26.5)**	
**Median age, years (range)**	**51 (25-70)**				
**cT status:**			0.471^b^		0.659^b^
cT0-cT3	187	37/183 (20.2)		36/132 (27.3)	
cT4	49	12/48 (25.0)		7/30 (23.3)	
**cN status**			0.654^b^		0.203^b^
cN0	98	19/95 (20.0)		15/69 (21.7)	
cN1	104	21/103 (20.4)		20/74 (27.0)	
cN2-4	34	9/33 (27.3)		8/19 (42.1)	
**M status**			0.162^b^		0.109ª
M1	25	8/25 (32.0)		6/13 (46.2)	
M0	211	41/206 (19.9)		37/149 (24.8)	
**Hormone receptor status**					
Estrogen receptor			0.332ª		0.816ª
ER-pos	133	32/131 (24.4)		27/101 (26.7)	
ER-neg	98	16/95 (16.8)		16/58 (27.6)	
Unknown	5				
Progesterone receptor			0.072ª		0.478ª
PgR-pos	113	30/110 (27.3)		26/88 (29.5)	
PgR-neg	118	18/116 (15.5)		16/71 (22.5)	
Unknown	5				
**Histological grade**			0.666^b^		0.960ª
Grade 1	14	4/13 (30.8)		3/12 (25.0)	
Grade 2	98	21/96 (21.9)		20/72 (27.8)	
Grade 3	110	22/108 (20.4)		18/72 (25.0)	
Unknown	14				
**Response**			1.000ª		0.784ª
Complete response	10	2/10 (20.0)		1/6 (16.7)	
Partial response	108	22/104 (21.2)		19/79 (24.1)	
Stable disease	88	18/87 (20.7)		15/61 (24.6)	
Progression	6	1/6 (16.7)		1/2 (50.0)	
Unknown	24				
**BM1 status (pretreatment)**					0.077^b^
Positive	49			14/37 (37.8)	
Negative	182			28/121 (23.1)	
Unknown	5				
**BM2 status (at surgery)**			1.000ª		0.654ª
Positive	11	2/11 (18.2)		1/7 (14.3)	
Negative	58	9/55 (16.4)		12/38 (31.6)	
Unknown	167				
**BM3 status (at 1 year follow-up)**			0.077^b^		
Positive	43	14/42 (33.3)			
Negative	119	23/116(19.8)			
Unknown	74				
**PB1 status (pretreatment)**			0.033ª		0.464ª
Positive	10	5/10 (50.0)		1//2 (50.0)	
Negative	209	39/204 (19.1)		39/148 (26.4)	
Unknown	17				
**PB2 status (at surgery)**			1.000ª		0.298ª
Positive	1	0/1 (0)		1/1 (100.0)	
Negative	70	12/67 (17.9)		13/46 (28.2)	
Unknown	165				
**PB3 status (at 1 year follow-up)**			0.618ª		0.004ª
Positive	6	2/6 (33.3)		5/6 (83.3)	
Negative	135	29/131 (22.1)		30/133 (22.6)	
Unknown	95				
**Relapse^c^**			0.642^b^		0.005^b^
Yes	98	22/98 (22.4)		22/55 (40.0)	
No	112			21/107 (19.6)	
**Relapse type**					
Locoregional only	4	0/4 (0)		0/2 (0)	
Distant metastases (skeletal and/or visceral)	76	18/74 (24.3)		15/42 (35.7)	
Distant metastases and locoregional	18	4/18 (22.2)		7/11 (63.6)	
**Death and cause^e^**					
Breast cancer death	106	23/105 (21.9)		26/53 (49.1)	
Any death	111	25/110 (22.7)		27/57 (47.4)	
Alive	124	24/120 (20.0)		16/105 (15.2)	

ª*P*-value computed by Fisher's exact test. ^b^*P*-value computed by Pearson Chi-square. ^c^Relapse data only for M0. ^d^The denominator (number assessed) for all fractions in the table represents the number of patients with known DTC status (positive or negative) for the respective parameter. ^e^No information available from one patient due to non-adherence.cT: clinical tumor status, cN: clinical lymph node status, M status: metastases status, DTC: disseminated tumor cells, BM: bone marrow, PB: peripheral blood.

### Association between DTC or CTC and clinical endpoints

The presence of ≥ 1 DTCs was not associated with tumor response to chemotherapy at the time of diagnosis, at the time of surgery, or 12 months after the start of NACT (Table 1). Clinical outcomes were registered for 12 years from study start or 10 years maximal follow-up. Ninety-eight of the M0 patients (46.4%) experienced a relapse. Out of these, 94 had a distant relapse, including 18 with both distant and locoregional relapse (Table 1). Breast cancer death was observed in 106 patients (including 20 patients with limited distant metastases at the time of inclusion (M1 patients).

**Figure 2:**
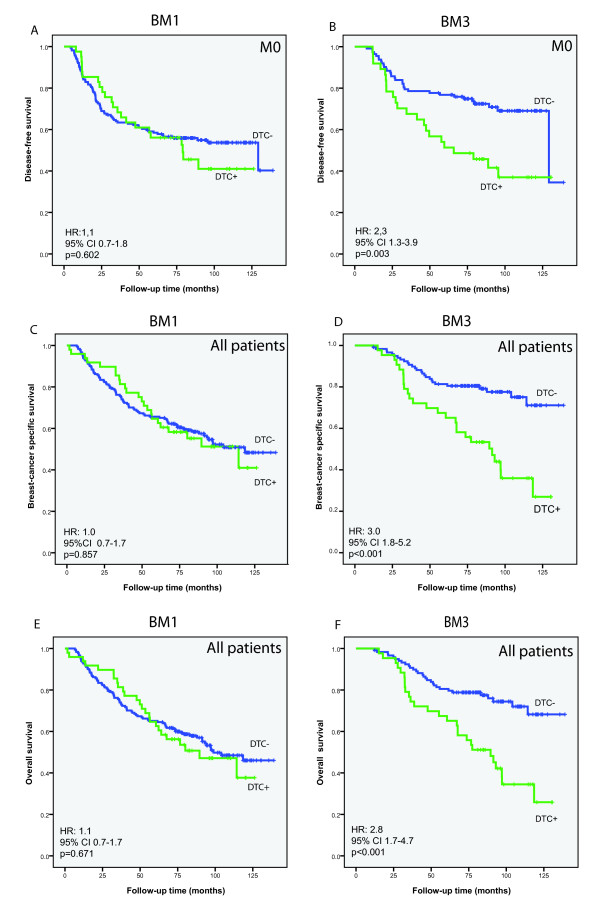
**Survival analyses according to disseminated tumor cell (DTC) status**. Kaplan-Meier plots for disease-free survival (DFS) (**A**), breast cancer specific survival (BCSS) **(C**) and overall survival (OS) (**E**) according to DTC status pretreatment (bone marrow aspiration 1 (BM1). DFS (**B**), BCSS (**D**) and OS (**F**) according to DTC status 12 months after start of neoadjuvant therapy (BM3). DTC+, ≥ 1 cytokeratin-positive cell per 2 × 10^6 ^MNC. DTC-, no cytokeratin-positive cells detected.

Table 2 presents the univariate survival analyses according to DTC/CTC status and primary tumor characteristics. Presence of ≥ 1 DTCs at BM1 did not have any influence on DFS among the M0 patients (Figure 2). Also, there was no association between the presence of DTCs at BM2 and clinical outcomes (Table 2). However, presence of ≥ 1 DTC at BM3 predicted reduced DFS (*P *= 0.003), BCSS (*P *< 0.001), and OS (*P *< 0.001) as also shown in Figure 2. The analysis of BCSS and OS also included patients with limited M1 status. No difference in the results was observed after exclusion of these patients (25 and 13 of those analyzed at BM1 and BM3, respectively; BCSS according to DTC status at BM3: hazard ratio (HR) 2.6, *P *= 0.002). The presence of ≥ 1 CTC at PB1 was associated with BCSS (*P *= 0.046), but not DFS (*P *= 0.146) (Table 2). Presence of CTCs at PB2 and PB3 did not affect survival. Achievement of CR was not associated with clinical outcomes (Table 2).

**Table 2:** Univariate survival analyses according to primary tumor factors and DTC/CTC status

	DFSª (*n *= 211)			BCSS^b ^(*n *= 236)			OS (*n *= 236)^c^		
	HR	95% CI	*P*	HR	95% CI	*P*	HR	95% CI	*P*
DTC status									
BM1-pos vs. BM1-neg	1.1	0.7-1.8	0.602	1.0	0.7-1.7	0.857	1.1	0.7-1.7	0.671
BM2-pos vs. BM2-neg	1.3	0.6-3.1	0.480	1.2	0.5-2.9	0.715	1.2	0.5-2.9	0.715
BM3-pos vs. BM3-neg	2.3	1.3-3.9	0.003	3.0^d^	1.8-5.2^d^	< 0.001^d^	2.8^e^	1.7-4.7^e^	< 0.001^e^
cN status (vs. cN0)			0.001			< 0.001			< 0.001
cN1	2.1	1.3-3.3	0.001	2.3	1.5-3.6	< 0.001	2.2	1.4-3.4	< 0.001
cN 2-4	2.5	1.4-4.5	0.003	3.0	1.7-5.3	< 0.001	2.8	1.6-4.8	< 0.001
Hormone receptor status									
PgR-pos vs. PgR-neg	1.6	1.0-2.3	0.034	2.0	1.4-2.8	< 0.001	1.8	1.3-2.6	0.001
ER-pos vs. ER-neg	1.7	1.2-2.6	0.007	1.9	1.3-2.6	< 0.001	1.8	1.3-2.5	0.001
Histological grade									
Grade 3 vs. grade 1-2	1.4	1.1-1.7	0.003	1.4	1.2-1.7	0.001	2.0	1.3-3.0	0.001
cT status									
cT4 vs. cTX-3	1.1	0.7-1.9	0.584	1.3	0.8-2.0	0.226	1.4	0.9-2.1	0.151
CTC status									
PB1-pos vs. PB1-neg	2.0	0.8-4.8	0.146	2.2^f^	1.0-4.8^f^	0.046^f^	2.1^g^	1.0-4.6^g^	0.057^g^
PB2 pos vs. PB2-neg	1.8	0.2-13.5	0.551	1.6	0.2-12.0	0.631	1.6	0.2-12.0	0.631
PB3-pos vs. PB3-neg	1.6	0.5-5.1	0.434	2.0	0.6-6.4	0.258	1.8	0.6-5.9	0.318
Clinical response									
No CR vs. CR	1.2	0.5-2.9	0.737	1.2	0.5-3.0	0.651	1.2	0.5, 2.9	0.727

ªDisease-free survival (DFS) only for M0 patients.

^b^Breast cancer specific survival (BCSS) for all patients.

^c^Overall survival (OS) for all patients.

^d^BCSS according to bone marrow aspiration at BM3 analyzed for M0 patients only: hazard ratio (HR) 2.6, 95% CI 1.4-4.6, *P *= 0.002.

^e^OS according to BM3 status analyzed for stage M0 patients only: HR 2.4, 95% CI 1.4-4.2, *P *= 0.003.

^f^BCSS according to peripheral blood sampling at PB1 analyzed for stage M0 patients only: HR 2.1, 95% CI 0.8-5.1, *P *= .116.

^g^OS according to PB1 status analyzed for stage M0 patients only: HR 2.0, 95% CI 0.8-4.9, *P *= 0.142.

BM1, BM2 and BM3: bone marrow aspiration pre-treatment, at surgery, and 12 months after start of neoadjuvant treatment respectively.

cT: clinical tumor status; cN: clinical lymph node status; M0: no metastases present; DTC: disseminated tumor cells; CTC: circulating tumor cells; CR: complete response.

### Multivariate survival analysis

Factors associated with prognosis on univariate analysis (Table 2) were included in a multivariate analysis (Table 3). DTC status at BM3 remained a significant prognostic factor for both DFS (HR 2.2, *P *= 0.005), BCSS (HR 2.6, *P *= 0.002) and OS (HR 2.6, *P *= 0.002), besides lymph node (N) status.

**Table 3:** Multivariate survival analyses

	DFSª			BCSS^b^			OS^b^		
	HR	95% CI	*P*	HR	95% CI	*P*	HR	95% CI	*P*
BM3-pos vs. BM3-neg	2.2	1.3-3.9	0.005	2.6	1.4-4.7	0.002	2.6	1.4-4.4	0.002
cN status (vs. N0)			0.053			0.032			0.047
cN1	2.0	1.0-3.8	0.040	2.3	1.1-4.8	0.019	2.2	1.1-4.3	0.024
cN 2-4	2.4	1.0-5.6	0.034	2.7	1.1-6.6	0.027	2.4	1.0-5.6	0.049
PgR-pos vs. PgR-neg	1.3	0.7-2.7	0.429	1.8	0.9-3.8	0.118	1.5	0.8-3.1	0.239
ER-pos vs. ER-neg	0.9	0.4-2.0	0.796	1.0	0.5-2.4	0.919	0.9	0.4-2.1	0.897
PB1-pos vs. PB1-neg				2.2	0.3-19.1	0.482	2.2	0.3-17.8	0.496
Grade 3 vs.grade 1-2	1.1	0.8-1.5	0.648	1.1	0.6-2.3	0.703	1.3	0.7-2.5	0.482

ªDisease-free survival (DFS) analyzed only in stage M0 patients.

^b^Breast cancer-free survival (BCSS) and overall survival (OS) analyzed in all patients.

Pos: positive; neg: negative; BM3: bone marrow aspiration pretreatment; cN: clinical node status; PgR: progesterone receptor; ER: estrogen receptor; PB1: peripheral blood sampling pretreatment.

### Other subgroup analyses

**Figure 3:**
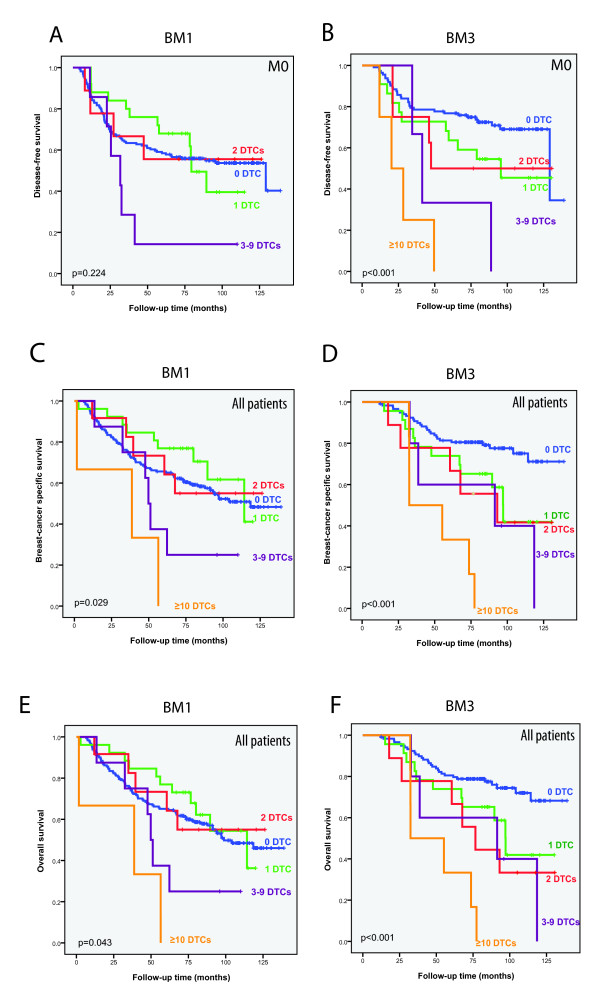
**Survival analyses according to disseminated tumor cell (DTC) number present**. Kaplan-Meier plots for disease-free survival (DFS), breast cancer specific survival (BCSS) and overall survival (OS) at the time of diagnosis/pretreatment according to number of DTCs detected pretreatment (bone marrow aspiration 1 (BM1) (**A**, **C**, **E**) and BM3 (**B**, **D**, **F**). In the Kaplan-Meier plot shown in Figure 3A, no patients had ≥ 10 DTCs.

Patients with DTCs were grouped according to the number of DTCs present (Figure 3). The results showed that patients with ≥ 10 DTCs at BM3 had detrimental DFS, BCSS and OS. Also, patients with 1, 2, or 3 to 9 DTCs experienced markedly reduced survival compared to those with no DTCs (*P *< 0.001). There were no significant outcome differences between these DTC-positive subgroups for BCSS and OS. At BM1, presence of 3 to 9, or ≥ 10 DTCs negatively affected survival (Figure 3). Comparing patients with 0 to 2DTCs versus ≥ 3 DTCs, a significant difference in DFS (HR 2.4, 95% confidence interval (CI) 1.03 to 5.4, *P *= 0.043) and in BCSS (HR 2.4, 95% CI 1.2 to 4.9, *P *= 0.011) was observed.

**Figure 4:**
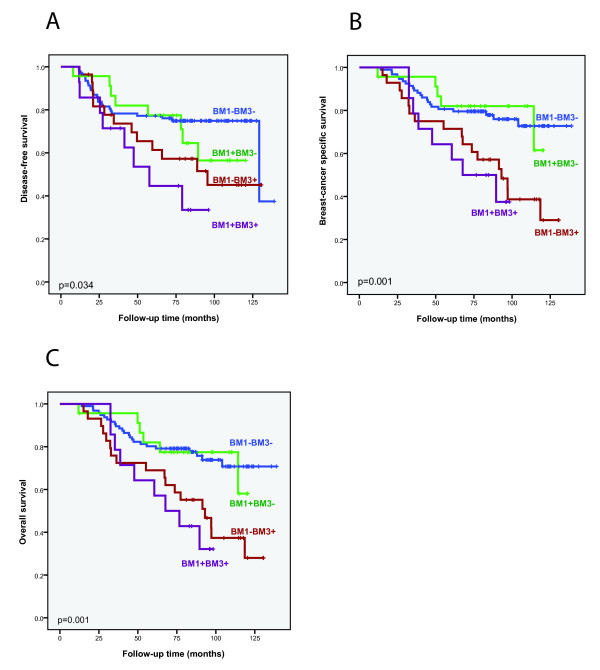
**Survival analyses according to the combined results of bone marrow (BM) aspiration performed pretreatment (BM1) and 12 months after start of neoadjuvant treatment (BM3)**. Kaplan-Meier plots for disease-free survival (DFS), breast cancer specific survival (BCSS) and overall survival (OS) according to the results of the combination of bone marrow disseminated tumor cell (DTC) status at BM1 and BM3.

To disclose the clinical significance of the DTC status at different time points, we combined the results from BM1 and BM3 (Figure 4). BM2 results were not included in this analysis, because of the relatively low number of patients with DTC status available at this time point. The results showed that presence of DTCs at BM1 only was unfavorable if DTCs persisted at BM3. Patients with a switch from positive to negative had similar prognosis to those with no DTC present at both time points. Regarding the CTC status, the positivity rate was too low for further subgroup analyses.

The number of patients analyzed for DTCs was reduced from 231 at BM1 to 162 at BM3 due to a combination of patient refusal or unsuccessful BM aspiration (*n *= 39), or relapse or death (*n *= 34) before this time point. One patients was excluded because of refusal to undergo postoperative radiotherapy (*n *= 1). We therefore performed additional survival analyses according to BM1 status for patients with no available DTC result at BM3. The data showed no survival difference between the DTC-positive (≥ 1 cell) and DTC-negative group (DFS: HR 0.9, 95% CI 0.4 to 2.1, *P *= 0.823 and BCSS: HR 1.2, 95% CI 0.6 to 2.3, *P *= 0.657).

## Discussion

In this study, we investigated the long-term prognostic impact of DTCs and CTCs before and after NACT in 236 patients with locally advanced breast cancer. We found the presence of DTCs 12 months from the start of NACT to be associated with a significantly elevated risk for systemic relapse and death in univariate as well as in multivariate analyses. Our results are consistent with the results of trials evaluating DTCs in the BM at follow-up in primary operable early breast cancer, including one smaller study performed six months after chemotherapy [4,7]. These results highlight the potential importance of including BM analyses as part of the follow-up for breast cancer patients in general, and in particular, for patients with locally advanced disease with their increased risk of relapse.

According to our data, there was no association between the DTC status before treatment and survival, when the threshold of positivity was set at ≥ 1 DTC. This is not in line with what other studies have shown [3]. This difference could be explained by the fact that all the patients included in our study were in a high-risk group, in contrast to the earlier stage breast cancer patients included in most of the published reports so far. Locally advanced breast cancer patients might have other biological factors influencing their prognosis. However, with a cutoff of ≥ 3 DTCs, DTC status at BM1 affected both DFS and BCSS. This indicates that patients with higher DTC loads were not sufficiently treated by the given chemotherapy. Moreover, patients with disappearance of DTCs between BM1 and BM3 clearly show improved survival, compared to patients with persisting DTCs. This is probably due to the fact that DTC results at BM1 does not account for the influence of chemotherapy, further emphasizing the importance of performing DTC analyses after completion of (and during) chemotherapy. Also, the switch from negativity at BM1 to positivity at BM3, clearly results in a worse prognosis, in line with patients being both positive at BM1 and BM3. These observations can be explained by the presence of low number of therapy-resistant DTCs (either below or above the detection limit), which will be detected as persistent DTCs at follow-up. This is also supported by an earlier publication from our group [5].

Decisions about systemic treatment are increasingly based on more detailed knowledge of the primary tumor characteristics like amplification or over-expression of HER2 [29] and hormone receptor positivity [30], which improves the patient outcomes. HER2 status was not analyzed, as the current study was performed prior to implementation of trastuzumab treatment and thus, routine HER2 testing, in the primary setting. However, with up-to-date systemic treatment we still experience only about 40 to 60% relative reduction in relapse. This indicates, especially in high risk groups, that a large number of patients still are insufficiently treated. As DTC status identifies a high risk group after completion of chemotherapy, DTC analysis during follow-up may open opportunities for introducing additional treatment. Hence, clinical trials should be designed to investigate the effect of secondary adjuvant treatment for DTC-positive patients in this window of opportunity with no overt metastases, only the presence of isolated tumor cells.

Pathological complete response (pCR) is a predictor of improved outcome in patients with locally advanced breast cancer [18]. In the current study, CR was not associated with improved survival, probably due to statistical limitations caused by the low CR rate achieved with the chemotherapy regimens. In addition, the use of clinical response criteria might affect the association between response and clinical outcome. Furthermore, no association between the detection of DTCs after NACT and CR was found (Table 1), which is in agreement with other published studies [31]. Again, the low CR rate might influence the possibility to show association between DTC status and CR. No significant difference in the frequency of DTC positivity was observed between BM1 and BM3. There was a trend towards association between DTC presence at BM1 and BM3 (*P *= 0.09) (Table 1). For the patients that had both a conclusive BM1 and BM2 result, we did not observe any change in the frequency of DTC positivity compared to BM1 (11 out of 66 positive at both time points) (Table 1). Others have performed serial BM aspiration, showing reduction in DTCs after systemic treatment, followed by increased positivity at later time points [32]. Different sensitivity in the DTC detection methods might cause differences between studies, and for the present study, the number of BM2 cases restricts the interpretation.

Our data, although limited by a low number of positive samples, also indicate that analyzing PB for CTCs at the time of diagnosis could identify high risk patients. This is in line with the results from other studies in early breast cancer [6,14,16]. However, CTC positivity was only associated with reduced BCSS and did not affect DFS (Table 2). As the analysis of BCSS (and OS) also included the stage M1 patients who were excluded from the DFS analysis, differences in the results might be expected. Indeed, analysis of BCSS without the M1 patients did not show a significant difference between CTC-positive and CTC-negative patients (Table 2, footnote). The low sensitivity of the present CTC analysis restricts further interpretation of the results. The study was performed with an ICC-based technique for detection of tumor cells both in bone marrow and peripheral blood, as this was the available and most standardized method at the time of the study initiation. The sample size used for detection of DTCs and CTCs was the same. As the concentration of CTCs is very low, increased sensitivity methods are probably needed. Today there are several technologies for CTC detection with higher sensitivity, including immunomagnetic separation, higher blood sample volumes and reverse transcriptase polymerase chain reaction (RT-PCR) [14,17,33]. It is likely that the number of CTCs detected in the samples from the patients in our study would have been higher if one of these technologies were utilized. Despite this, we observed an association between CTC and DTC presence both before NACT and one year after the start of NACT (Table 1). Similar findings have been published from other groups [34,35]. The prognostic impact of DTCs compared to CTCs varies between studies [6,35-37]. This could partly be explained by the discrepancies in the detection methods causing differences in sensitivity and specificity, and possibly different relevance of DTCs and CTCs depending on the breast tumor subtype [14]. DTCs and CTCs might possibly be two biologically different entities, the DTCs may stay dormant in the BM for several years, and the CTCs might be migrating from a more actively proliferating cell clone in the tumor. Hence, the DTCs could be expected to be more predictive of the risk for later development of metastases or relapse [35].

Molecular characterization of the primary tumor and the corresponding DTCs and CTCs is possible both at the protein, RNA and DNA level [38-40]. Recently, results from whole genome-based molecular characterization of single tumor cells, have also been reported [41,42]. These methods may provide information about the metastatic potential of the single tumor cell and their tumor initiating capacity, as well as characteristics of dormant tumor cells. The opportunity to characterize these cells opens possibilities for identification of markers which may guide treatment decisions [43].

## Conclusions

In conclusion, this study shows that for patients with locally advanced breast cancer, the presence of occult tumor cells in blood or bone marrow predicts poor clinical outcome. Especially, the persistence of DTCs after neoadjuvant therapy and surgery is a strong prognostic marker. Further characterization of DTCs is still needed, as well as improved biological understanding of what determines the presence of DTCs. This may lead to establishment of more efficient adjuvant therapies for DTC-positive patients in the future.

## Abbreviations

APAAP: alkaline phosphate/antialkaline phosphatase; BCSS: breast cancer specific survival; BM: bone marrow; BMA: bone marrow aspiration; CK: cytokeratin; CR: complete response; CTCs: circulating tumor cells; DFS: disease-free survival; DTCs: disseminated tumor cells; ER: estrogen receptor; HER2: human epidermal growth factor receptor 2; HR: hazard ratio; ICC: immunocytochemical; NACT: neoadjuvant chemotherapy; MNC: mononuclear cells; OS: overall survival; PB: peripheral blood; pCR: pathological complete response; PD: progressive disease; PgR: progesterone receptor; RT-PCR: reverse transcriptase polymerase chain reaction; SD: stable disease.

## Competing interests

The authors declare that they have no competing interests.

## Authors' contributions

RRM and BN were responsible for drafting the manuscript and RM participated in data collection and statistical analyses. EB and AR participated in the processing and analyses of DTCs and EB contributed to writing of the manuscript. EL, GA, BØ, SL, TR, and IM participated in the design of the study and included patients in the study. JMN was responsible for the interpretation of the DTC results. GK, PEL and BN were responsible for study design and PEL contributed to writing of the manuscript. BN was central in the statistical analyses, data interpretation and manuscript writing. All authors read and approved the final manuscript.
